# Genomic surveillance of multidrug-resistant Escherichia coli and Klebsiella in clinical and wastewater isolates from a paediatric hospital in Lima, Peru

**DOI:** 10.1099/acmi.0.001006.v3

**Published:** 2026-01-14

**Authors:** Brenda Ayzanoa, Camila Castillo-Vilcahuaman, Guillermo Salvatierra, Alejandra Dávila-Barclay, Diego Cuicapuza, Janet Huancachoque, Maritza Calderón, Emiliana Rizo-Patrón, Carlos Santillán-Salas, Robert H. Gilman, Pablo Tsukayama

**Affiliations:** 1 Laboratorio de Genómica Microbiana, Facultad de Ciencias e Ingeniería, Universidad Peruana Cayetano Heredia, Lima, Peru; 2 CCTE Ciencia y Vida, Fundación Ciencia y Vida, Huechuraba, Santiago, Chile; 3 Facultad de Ciencias de la Salud, Universidad Privada del Norte, Lima, Peru; 4 Facultad de Medicina, Universidad Peruana Cayetano Heredia, Lima, Peru; 5 Laboratorios de Enfermedades Infecciosas, Laboratorios de Investigación y Desarrollo, Universidad Peruana Cayetano Heredia, Lima, Peru; 6 Instituto Nacional de Salud del Niño San Borja, Lima, Peru; 7 Clínica Delgado Auna, Lima, Peru; 8 Johns Hopkins School of Public Health, Baltimore, MD, USA; 9 Instituto de Medicina Tropical Alexander von Humboldt, Universidad Peruana Cayetano Heredia, Lima, Peru; 10 Parasites and Microbes Programme, Wellcome Sanger Institute, Hinxton, UK

**Keywords:** antimicrobial resistance, enterobacteria, wastewater, whole-genome sequencing

## Abstract

The environmental spread of antibiotic-resistant bacteria is a growing global health concern, particularly in low- and middle-income countries where limited wastewater treatment infrastructure may facilitate the dissemination of multidrug-resistant (MDR) organisms. *Escherichia coli* and *Klebsiella* spp. are clinically significant MDR pathogens commonly associated with healthcare-associated infections and known to carry diverse antimicrobial resistance genes (ARGs). In this study, we conducted genomic and phenotypic analyses of *E. coli* and *Klebsiella* spp. isolated from hospital wastewater and paediatric patient samples at a tertiary hospital in Lima, Peru, between 2017 and 2019. A total of 157 isolates were collected (*E. coli*, *n*=113; *Klebsiella* spp., *n*=44). Whole-genome sequencing was performed to identify ARGs and assess sequence types (STs). MDR phenotypes were more prevalent among wastewater isolates (73.5%) compared to clinical isolates (56.8%, *P*=0.014), while extended-spectrum *β*-lactamase production was more frequent in clinical isolates (52.9 % vs. 13.9 %, *P*<0.001). Carbapenemase-producing isolates were found only in wastewater, whereas colistin resistance was restricted to a subset of clinical *E. coli* isolates from urine. Genomic analysis revealed greater sequence type diversity among wastewater isolates, including high-risk STs such as ST10, ST131 and ST405. The Shannon diversity index was higher for wastewater-derived isolates (H=3.45) compared to clinical isolates (H=2.95), indicating a more heterogeneous resistance reservoir. In total, 1,302 resistance gene hits were identified, with clinical isolates carrying significantly more ARGs per genome than wastewater isolates. A small number of shared STs were detected in both sources, suggesting possible overlap in bacterial populations. Our findings highlight the potential role of hospital wastewater as a reservoir of antimicrobial resistance and support the value of integrating environmental and clinical genomic surveillance. Wastewater-based monitoring may inform infection control efforts and guide interventions to curb the spread of AMR within healthcare settings and their surrounding environments.

## Data Summary

RAW sequencing reads (.fastq) generated in this study have been deposited in the NCBI database under BioProject ID PRJNA1168566. Individual sequence accession numbers are available in the supplementary table.

## Introduction

The increasing prevalence of antimicrobial resistance (AMR) is a significant global public health concern, particularly in low- and middle-income countries (LMICs), where limited wastewater treatment and infection control measures contribute to the persistence and dissemination of multidrug-resistant (MDR) bacteria [1,2]. Among the most clinically relevant MDR pathogens are *Escherichia coli* and *Klebsiella* spp., which frequently cause healthcare-associated infections such as urinary tract infections, bloodstream infections and pneumonia [3,5]. These pathogens are of particular concern due to their ability to acquire and disseminate resistance determinants, including extended-spectrum *β*-lactamases (ESBLs) and carbapenemases, which render many frontline antibiotics ineffective [6,8].

Hospital wastewater represents a potential reservoir for MDR bacteria and resistance genes, as it contains a mixture of human-associated microbes, antimicrobial residues and disinfectants that exert selective pressure, favouring the persistence of resistant strains [9,10]. Studies have shown that hospital effluents harbour clinically significant MDR Enterobacteriaceae, including carbapenem-resistant *Klebsiella* spp. and ESBL-producing *E. coli*, which can enter municipal sewage systems and contribute to the environmental dissemination of AMR [11,13]. In LMICs, where wastewater treatment infrastructure is often inadequate, these reservoirs may serve as a pathway for reintroducing resistant bacteria into clinical and community settings [14]. Despite this recognized risk, there is a lack of genomic studies comparing resistance profiles between hospital wastewater and clinical isolates in Peru, limiting our understanding of how hospital effluents contribute to the transmission of MDR bacteria.

Traditional molecular typing methods, such as PFGE and MLST, have been widely used to characterize bacterial strains in AMR surveillance but lack the resolution to fully capture the complexity of resistance gene transfer and bacterial evolution [15]. Whole-genome sequencing (WGS) provides a higher-resolution approach by enabling the comprehensive identification of antimicrobial resistance genes (ARGs), mobile genetic elements and phylogenetic relationships between strains [16,18]. WGS has become an essential tool for outbreak investigations and the characterization of emerging MDR clones in hospital settings, offering insights into the evolutionary dynamics of resistant pathogens [19,22]. Despite increasing recognition of hospital wastewater as a reservoir of AMR, few genomic studies in LMICs have systematically compared clinical and wastewater isolates. Understanding these genetic links is crucial for designing surveillance and intervention strategies.

This study provides a genomic and phenotypic characterization of *E. coli* and *Klebsiella* spp. isolated from hospital wastewater and clinical samples in a paediatric hospital in Lima, Peru, over a 2-year period over a 2-year period. By integrating WGS with antimicrobial susceptibility testing, we aim to evaluate the extent to which wastewater contributes to the dissemination of MDR bacteria and resistance genes within hospital and community settings. Our findings will contribute to the growing body of evidence supporting the use of genomic surveillance as a tool for AMR monitoring in both clinical and environmental contexts, particularly in resource-limited settings where AMR control strategies remain a critical public health challenge [23].

## Methods

### Study site and sample collection

This study was conducted at the Instituto Nacional de Salud del Niño San Borja (INSN-SB), a national referral paediatric hospital in Lima, Peru. The hospital provides specialized care in neonatal surgery, cardiology and burn treatment and has implemented an Antibiotic Stewardship Programme to mitigate antimicrobial resistance [24]. Despite these efforts, there is limited data on resistant bacteria in hospital wastewater. Hospital wastewater samples (*n*=26) were collected from the central sewer system at two locations between October 2017 and May 2019 over 14 sampling dates. Samples were collected in sterile 50-ml conical centrifuge tubes, stored at 4 °C, and transported to the laboratory following national sanitary guidelines [25]. Clinical isolates of *E. coli* (*n*=34) and *Klebsiella* spp. (*n*=15) were obtained from urine and blood cultures per paediatric patient admitted to the intensive care unit (ICU) at INSN-SB between December 2018 and May 2019. These isolates were identified using the BD Phoenix automated identification system (Becton Dickinson, Franklin Lakes, NJ) as part of routine clinical diagnostics.

### Bacterial isolation and antimicrobial susceptibility testing

Wastewater samples were centrifuged at 10,000 r.p.m. for 10 min, and the pellet was streaked onto CHROMagar Orientation medium and incubated at 37 °C for 24 h. Colonies were subcultured on MacConkey agar for lactose fermentation confirmation. Biochemical identification was performed using triple sugar iron agar, motility indole, ornithine, lysine, iron agar and Simmons’ citrate agar tests [3]. Antimicrobial susceptibility testing was conducted according to the Clinical and Laboratory Standards Institute 2018 guidelines [26]. Twenty-one antibiotics were tested using the disc diffusion method (Table 1). Isolates resistant to at least three antibiotic classes were classified as MDR [27]. ESBL production was confirmed using the combination disc test with cefotaxime, aztreonam, ceftazidime and cefepime placed around an amoxicillin-clavulanic acid disc, following EUCAST 2024 guidelines [28]. AmpC *β*-lactamase production was determined using cefoxitin as an inducer, while carbapenemase activity was assessed using imipenem and meropenem discs with EDTA and phenylboronic acid inhibitors to differentiate between metallo-*β*-lactamases and serine carbapenemases [29]. *E. coli* ATCC 25922 was used as a quality control strain throughout susceptibility testing.

**Table 1.** Resistance profiles and bivariate analysis of *E. coli* isolates from clinical and wastewater samples

Result	*E. coli* (*n*=113)					
	Total	Wastewater (*n*=79)	Clinical samples (*n*=34)		Total	*P*-value*
			Urine (*n*=30)	Blood (*n*=4)		
*Multidrug resistance*						
Positive	64 (56.6)	39 (69.9)	22 (73.3)	3 (75.0)	25 (73.5)	0.014
*ESBL*						
Positive	29 (25.6)	11 (13.9)	14 (46.7)	4 (100)	18 (52.9)	<0.001
*AmpC*						
Positive	3 (2.7)	3 (3.8)	0 (0)	0 (0)	0 (0)	0.338
*Carbapenemases*						
Positive	1 (0.9)	1 (1.26)	0 (0)	0 (0)	0 (0)	0.699
*Polymyxins*						
Colistin	5 (4.4)	0 (0)	5 (16.6)	0 (0)	5 (14.7)	0.002
*Phenicols*						
Chloramphenicol	22 (19.5)	13 (16.5)	8 (26.7)	1 (25.0)	9 (26.5)	0.165
*Tetracyclines*						
Tetracycline	53 (46.9)	32 (40.5)	18 (60.0)	3 (75.0)	21 (61.8)	0.031
*Sulphonamides*						
Trimethoprim/sulfamethoxazole	52 (46.0)	33 (41.7)	18 (60.0)	1 (25.0)	19 (55.9)	0.120
*Aminoglycosides*						
Gentamicin	18 (15.9)	14 (17.7)	3 (10.0)	1 (25.0)	4 (11.8)	0.311
Amikacin	2 (1.8)	2 (2.5)	0 (0)	0 (0)	0 (0)	0.487
*Macrolides*						
Azithromycin	27 (23.9)	16 (20.3)	11 (36.7)	0 (0)	11 (32.4)	0.127
*Penicillins*						
Amoxicillin	69 (61.1)	41 (51.9)	24 (80.0)	4 (100)	28 (82.4)	0.002
Amoxicillin with clavulonic acid	8 (7.1)	7 (8.9)	1 (3.3)	0 (0)	1 (2.9)	0.244
Aztreonam	17 (15.0)	7 (8.9)	7 (23.3)	3 (75.0)	10 (29.4)	0.007
Piperacillin	63 (55.8)	36 (45.6)	23 (76.7)	4 (100)	27 (79.4)	0.001
*Cephalosporins*						
Cefalotin	37 (32.7)	16 (20.3)	18 (60.0)	3 (75.0)	21 (61.8)	<0.001
Cefotaxime	31 (27.4)	11 (13.9)	17 (56.7)	3 (75.0)	20 (58.8)	<0.001
Cefepime	18 (15.9)	6 (7.6)	9 (30.0)	3 (75.0)	12 (35.3)	0.001
Ceftazidime	13 (11.5)	6 (7.6)	4 (13.33)	3 (75.0)	7 (20.6)	0.052
Ceftriaxone	30 (26.5)	11 (13.9)	16 (53.33)	3 (75.0)	19 (55.9)	<0.001
Cefoxitin	4 (3.5)	4 (5.1)	0 (0)	0 (0)	0 (0)	0.233
*Carbapenemases*						
Meropenem	1 (0.9)	1 (1.3)	0 (0)	0 (0)	0 (0)	0.699
Imipenem	1 (0.9)	1 (1.3)	0 (0)	0 (0)	0 (0)	0.699
*Quinolones*						
Nalidixic acid	57 (50.4)	31 (39.2)	22 (73.3)	4 (100)	26 (76.5)	<0.001
Ciprofloxacin	25 (22.1)	8 (10.1)	13 (43.3)	4 (100)	17 (50.0)	<0.001

* P*-value: calculated using Fisher’s exact test with a 95% confidence level

AmpC, AmpC β-lactamase production; Carbapenemases, carbapenemase production; ESBL, extended-spectrum Beta-Lactamase production.

### WGS and bioinformatic analysis

Genomic DNA was extracted from 1 ml of overnight cultures using the GeneJET Genomic DNA Purification Kit (Thermo Scientific, Waltham, MA). Libraries were prepared using the Illumina Nextera XT kit with 1 ng of DNA and sequenced on an Illumina MiSeq platform (2×250 paired-end reads) using the MiSeq V2 Reagents Kit (500 cycles), yielding an average sequencing depth of 84× (range: 17×–163×). The average sequencing coverage was 84× (range: 17×–163×). Raw sequencing reads were assessed for quality using FastQC v0.72 and adapters were removed with Fastp v0.23.4 (https://github.com/OpenGene/fastp). Reads were assembled de *novo* using SPAdes v3.12.0 [30] and evaluated with Quast v5.2.0 [31], with genomes below an N50 threshold of 16,000 bp excluded. Genome quality was evaluated with CheckM v1.2.4 (https://github.com/Ecogenomics/CheckM), and only assemblies with completeness >95% and contamination <5% were retained; in addition, genomes with coverage below 20× were excluded. MLST was performed using MLST v2.22.0 using the *E. coli* ecoli_achtman_4 scheme, while *Klebsiella* spp. were typed with Kleborate v2.2.0 (https://github.com/klebgenomics/Kleborate) [32,33]. Novel alleles were submitted to EnteroBase (https://enterobase.warwick.ac.uk/) for the correct assignment of new sequence types, and ARGs were identified using Abricate v1.0.1 with the National Center for Biotechnology Information (NCBI) database (release downloaded: 27 March 2021) [34], and genome annotation was performed with Prokka v1.14.6. The resulting GFF files were then used for the pangenome reconstruction and the core genome alignment using Roary v3.13 [35], and phylogenetic trees were inferred using RAxML v8.2.12 [36] under the GTR+GAMMA substitution model with 100 bootstrap replicates. Finally, trees were visualized and annotated with iTOL v7 [37].

### Diversity and statistical analysis

Genomic diversity among *E. coli* isolates was assessed using the Shannon diversity index, a standard metric for evaluating phylogenetic richness within bacterial populations implemented with the vegan package (v2.7–1) in R v4.5.0 [38,39]. Statistical comparisons of resistance rates were performed using Fisher’s exact test in STATA 16 (StataCorp, College Station, TX), applying a 95% confidence level. For all statistical comparisons, *P*-values lower than 0.05 were considered significant.

## Results

### Antimicrobial susceptibility and resistance profiles

**Fig. 1.**
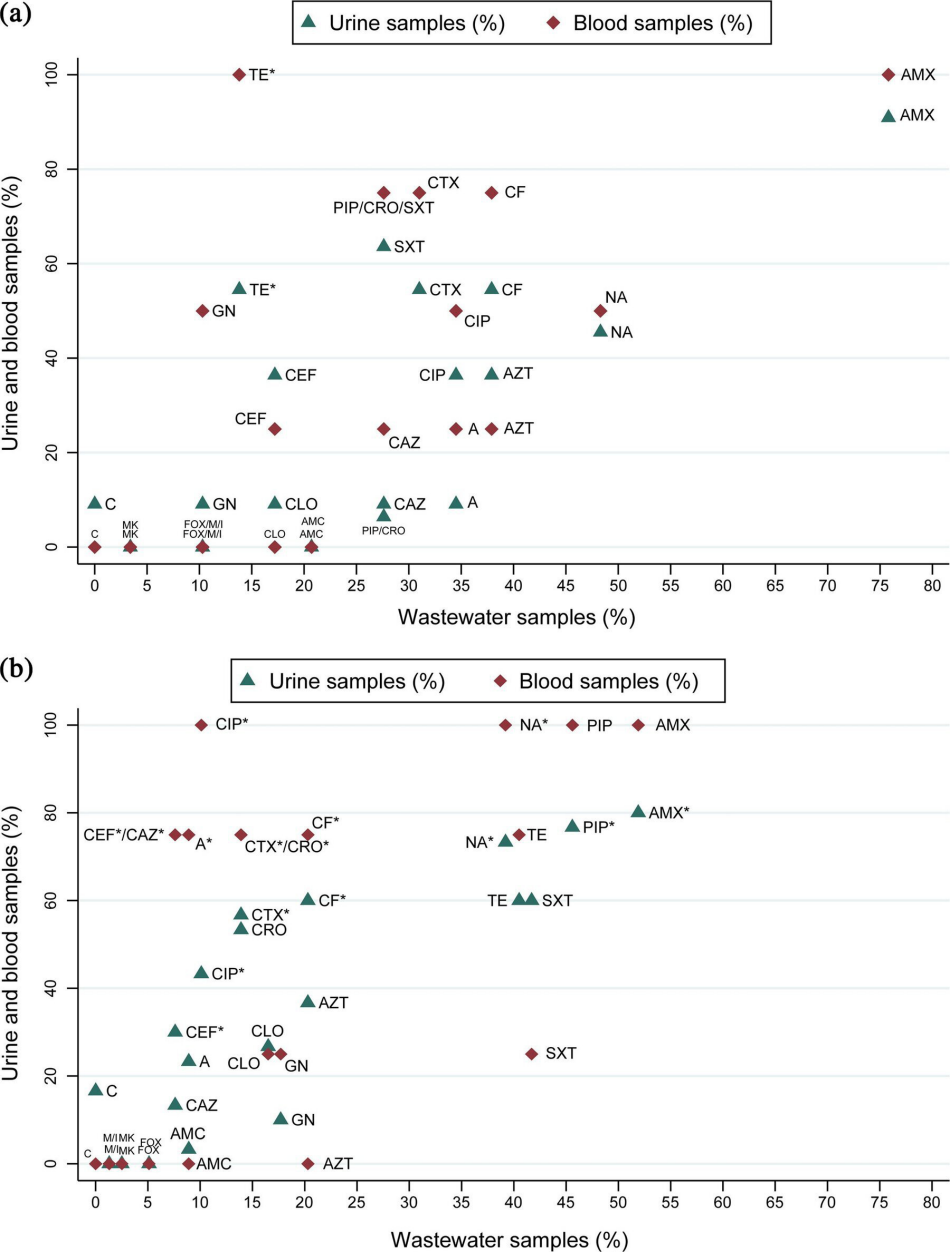
Antibiotic Antimicrobial resistance rates in *Klebsiella* spp*.* (**a**) and *E. coli* (**b**) isolates from hospital wastewater and clinical samples. Resistance rates in hospital wastewater isolates (*n*=113) are compared with clinical isolates from urine (*n*=11) and blood (*n*=4) collected from the same hospital. Asterisks (*) indicate statistically significant differences (*P*<0.05, Fisher’s exact test) between blood/urine isolates and wastewater isolates. Abbreviations: TET, tetracycline; AMX, amoxicillin; SXT, trimethoprim/sulfamethoxazole; NA, nalidixic acid; CLO, chloramphenicol; CF, cefalotin; CIP, ciprofloxacin; CTX, cefotaxime; AZT, azithromycin; GN, gentamicin; AMC, amoxicillin–clavulanic acid; FEP, cefepime; CAZ, ceftazidime; C, colistin; FOX, cefoxitin; M, meropenem; I, imipenem; A, aztreonam; PIP, piperacillin; CRO, ceftriaxone; MK, amikacin.

One hundred fifty-seven isolates were analysed, including *E. coli* (*n*=113) and *Klebsiella* spp. (*n*=44), recovered from hospital wastewater (*n*=113) and clinical samples (urine, *n*=44; blood, *n*=9). Among *E. coli* isolates, the prevalence of MDR was significantly higher in clinical isolates (73.5%) compared to wastewater isolates (69.9%) (*P*=0.014, Fisher’s exact test). ESBL production was also more frequent in clinical isolates (52.9%) than in wastewater isolates (13.9%, *P*<0.001). Carbapenemase production was observed exclusively in wastewater *E. coli* isolates (1.26%, *n*=1), whereas colistin resistance was only detected in clinical urine isolates (16.6%, *n*=5) (Table 1). Among *Klebsiella* spp*.* isolates, MDR phenotypes were detected more frequently in clinical isolates (60.0%) than in wastewater isolates (48.3%), though this difference was not statistically significant (*P*=0.338). ESBL production was significantly higher in clinical isolates (60.0%) than in wastewater isolates (24.1%) (*P*=0.023). Notably, carbapenemase production (17.2%) and AmpC *β*-lactamase production (3.4%) were only detected in wastewater isolates. Resistance to amikacin, cefoxitin, meropenem and imipenem was found exclusively in wastewater isolates, whereas colistin resistance (9.1%) was observed only in one clinical urine isolate (Table 2). Resistance rates for key antibiotics varied between *E. coli* and *Klebsiella* spp*.* isolates from clinical and wastewater sources, with a higher prevalence of ESBL production in clinical isolates and carbapenem resistance detected exclusively in wastewater isolates (Fig. 1).

**Table 2.** Resistance profiles and bivariate analysis of *Klebsiella* spp. isolates from clinical and wastewater samples

Results	*Klebsiella* sp. (*n*=44)					
	Total	Wastewater (*n*=29)	Clinical samples (*n*=15)		Total	*P*-value*
			Urine (*n*=11)	Blood (*n*=4)		
*Multidrug resistance*						
Positive	23 (52.3)	14 (48.3)	6 (54.5)	3 (75.0)	9 (60.0)	0.338
*ESBL*						
Positive	16 (36.4)	7 (24.1)	6 (54.5)	3 (75.0)	9 (60.0)	0.023
*AmpC*						
Positive	1 (2.2)	1 (3.4)	0 (0)	0 (0)	0 (0)	0.659
*Carbapenemases*						
Positive	5 (11.4)	5 (17.2)	0 (0)	0 (0)	0 (0)	0.109
*Polymyxins*						
Colistin	1 (2.2)	0 (0)	1 (9.1)	0 (0)	1 (6.7)	0.341
*Phenicols*						
Chloramphenicol	6 (13.6)	5 (17.2)	1 (9.1)	0 (0)	1 (6.7)	0.320
*Tetracyclines*						
Tetracycline	14 (31.8)	4 (13.8)	6 (54.5)	4 (100)	10 (66.7)	0.001
*Sulphonamides*						
Trimethoprim/sulfamethoxazole	18 (40.9)	8 (27.6)	7 (63.6)	3 (75.0)	10 (66.7)	0.015
*Aminoglycosides*						
Gentamicin	6 (13.6)	3 (10.3)	1 (9.1)	2 (50.0)	3 (20.0)	0.327
Amikacin	1 (2.2)	1 (3.4)	0 (0)	0 (0)	0 (0)	0.659
*Macrolides*						
Azithromycin	16 (36.4)	11 (37.9)	4 (36.4)	1 (25.0)	5 (33.3)	0.516
*Penicillins*						
Amoxicillin	36 (81.8)	22 (75.8)	10 (90.9)	4 (100)	14 (93.3)	0.156
Amoxicillin with clavulonic acid	6 (13.6)	6 (20.7)	0 (0)	0 (0)	0 (0)	0.067
Aztreonam	12 (27.3)	10 (34.5)	1 (9.1)	1 (25.0)	2 (13.3)	0.127
Piperacillin	18 (40.9)	8 (27.6)	7 (6.4)	3 (75.0)	10 (66.7)	0.015
*Cephalosporins*						
Cefalotin	20 (45.5)	11 (37.9)	6 (54.5)	3 (75.0)	9 (60.0)	0.141
Cefotaxime	18 (40.9)	9 (31.0)	6 (54.5)	3 (75.0)	9 (60.0)	0.063
Cefepime	10 (22.7)	5 (17.2)	4 (36.4)	1 (25.0)	5 (33.3)	0.202
Ceftazidime	10 (22.7)	8 (27.6)	1 (9.1)	1 (25.0)	2 (13.3)	0.250
Ceftriaxone	18 (40.9)	8 (27.6)	7 (6.4)	3 (75.0)	10 (66.7)	0.015
Cefoxitin	3 (6.8)	3 (10.3)	0 (0)	0 (0)	0 (0)	0.276
*Carbapenems*						
Meropenem	3 (6.8)	3 (10.3)	0 (0)	0 (0)	0 (0)	0.276
Imipenem	3 (6.8)	3 (10.3)	0 (0)	0 (0)	0 (0)	0.276
*Quinolones*						
Nalidixic acid	21 (47.7)	14 (48.3)	5 (45.5)	2 (50.0)	7 (46.7)	0.586
Ciprofloxacin	16 (36.4)	10 (34.5)	4 (36.4)	2 (50.0)	6 (40.0)	0.484

*P*-value: calculated using Fisher’s exact test with a 95% confidence level.

AmpC, AmpC β-lactamase production ; Carbapenemases, carbapenemase production; ESBL, extended-spectrum Beta-Lactamase production.

### Genomic diversity and shared sequence types

**Fig. 2.**
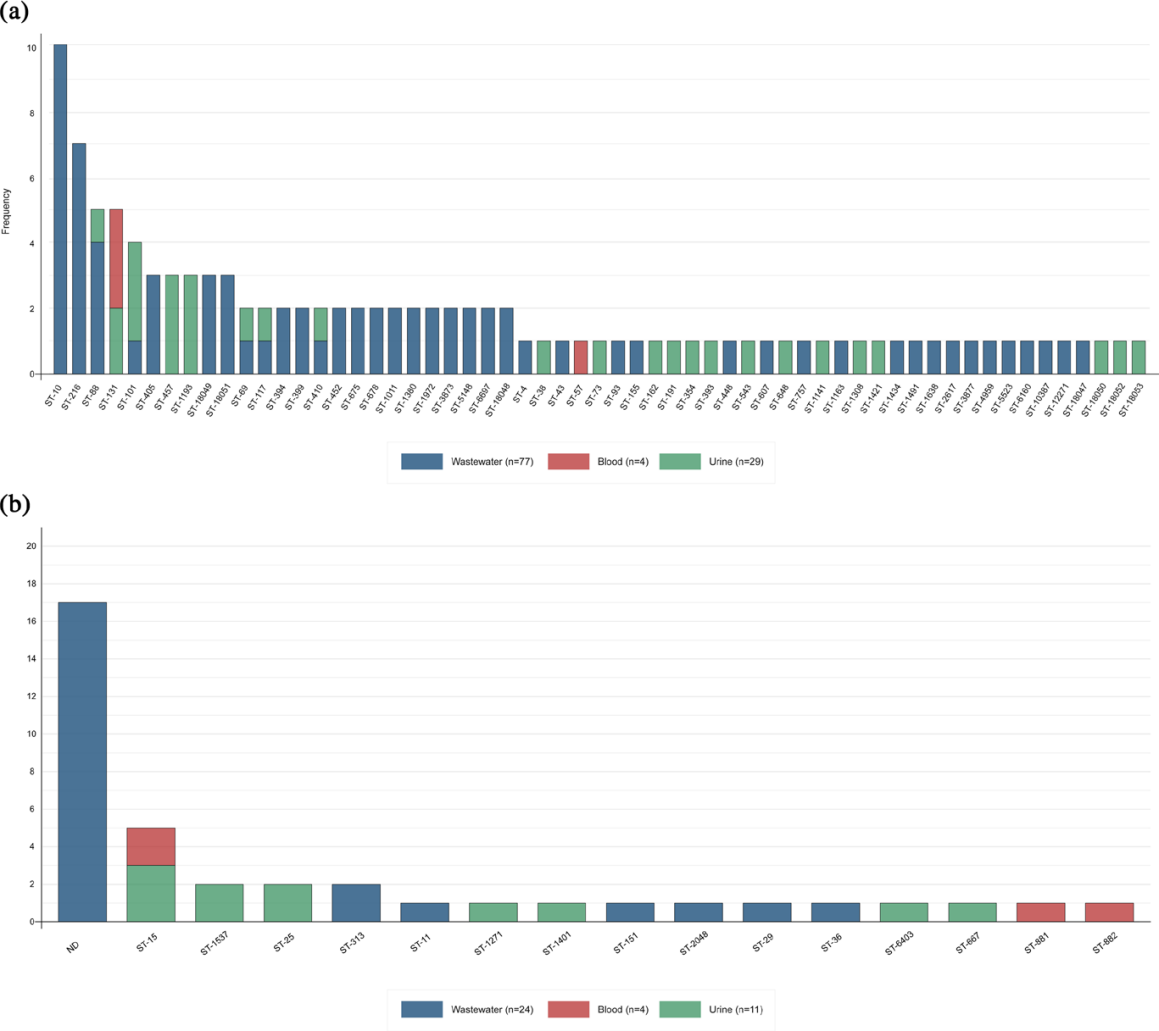
Distribution of STs among sequenced *E. coli* and *Klebsiella* spp*.* isolates. The upper panel represents *E. coli* sequence types, while the lower panel shows *Klebsiella* spp*.* sequence types. Colours indicate different sample sources (clinical or wastewater), highlighting the diversity of sequence types in each group.

Following bioinformatic quality control, 39 *Klebsiella* and 110 *E. coli* isolates were retained for analysis. Whole-genome sequencing identified 59 distinct sequence types (STs) among *E. coli* and 16 among *Klebsiella*, reflecting substantial genetic diversity across clinical and wastewater sources. Among clinical *E. coli*, the most frequent STs were ST131 (*n*=5), ST101 (*n*=3), ST1193 (*n*=3) and ST457 (*n*=3), consistent with their global association with hospital-acquired infections. In contrast, wastewater isolates were dominated by ST10 (*n*=10), ST216 (*n*=7) and ST88 (*n*=4). ST88 and ST101 were recovered from both clinical and wastewater samples, suggesting overlapping reservoirs of potentially pathogenic strains (Fig. 2). Many *Klebsiella* isolates from wastewater could not be confidently assigned to a definitive ST; the predominant species within this group were *Klebsiella michiganensis* (*n*=10), *Klebsiella grimontii* (*n*=6) and *Klebsiella pasteurii* (*n*=1). Among clinical *Klebsiella pneumoniae* isolates, the most common ST was ST15 (*n*=5).

**Fig. 3.**
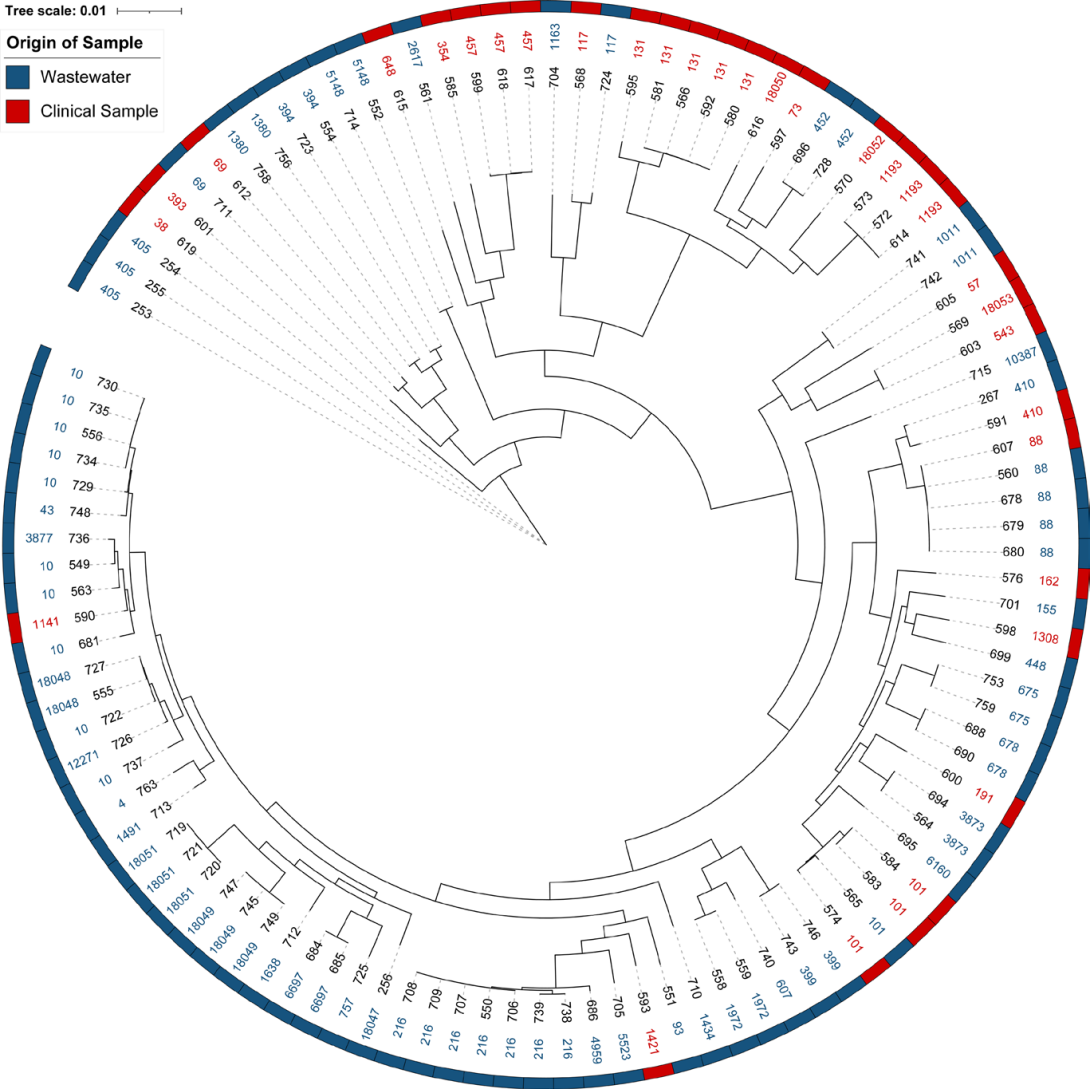
Core genome phylogeny of *E. coli* isolates from hospital wastewater and clinical sources. Isolates are labelled by sequence identifier (black text), and STs are colour-coded. Coloured strips adjacent to the tree indicate sample origin (wastewater vs. clinical). The tree shows distinct clustering of most clinical and wastewater isolates, while certain shared STs formed closely related clusters regardless of source, consistent with potential overlap between hospital- and wastewater-associated bacterial populations.

Phylogenetic analysis showed that clinical and wastewater isolates generally clustered separately (Fig. 3), although some overlapping STs formed closely related clusters irrespective of origin, supporting possible bacterial flow between environments. The Shannon diversity index was higher in wastewater isolates (H=3.45) compared to clinical isolates (H=2.95), consistent with wastewater representing a more diverse reservoir of resistance determinants, likely shaped by continuous bacterial input from hospital and environmental sources. Seven novel STs were identified, six from wastewater [including ST18047 and ST18048 within clonal complex (CC) 10, and ST18051 (CC165) and ST18049 (CC226)] and one from clinical isolates (ST18050, CC95).

### Resistome analysis

Nine hundred ninety-four ARG hits representing 109 unique genes were identified across all isolates. Clinical *E. coli* isolates carried a significantly higher average number of ARGs (7.84, 95 % CI 6.53–9.14) compared to wastewater isolates (4.85, 95 % CI 3.91–5.79) (*P*=0.0006). Similarly, clinical *Klebsiella* isolates harboured more ARGs (11.2, 95 % CI 8.58–13.81) than wastewater isolates (8.58, 95 % CI 6.28–10.88) (*P*=0.1463).

**Fig. 4.**
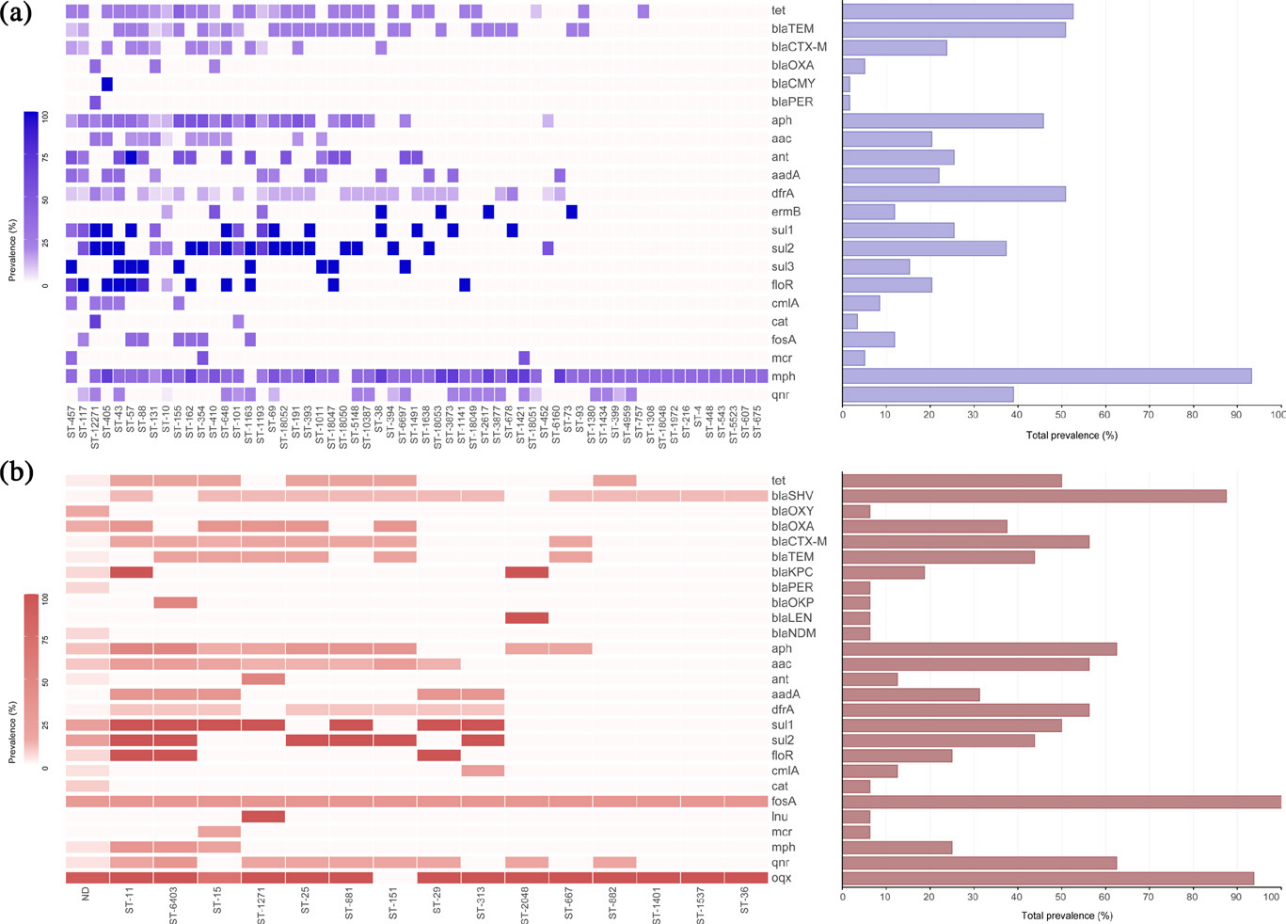
Prevalence of ARGs across STs in *E. coli* (**a**) and *Klebsiella* spp. (**b**).

In *E. coli*, the *mphB* gene (85.84%, *n*=94) was the most prevalent ARG, while ST-131 isolates carried multiple resistance genes, including ${bla}_{OXA-1}$ (*n*=4),${bla}_{CTX-M-15}$ (*n*=3) and ${bla}_{TEM-1}$ (*n*=1). Colistin resistance genes (*mcr-1*) were detected exclusively in urine isolates of ST-1421 (*n*=1), ST-457 (*n*=2) and ST-354 (*n*=1) (Fig. 4). Among *Klebsiella* isolates, *oqxA* (*n*=36, 92.3%) was present in almost all genomes, with *K. pneumoniae* ST-15 frequently carrying ${bla}_{SHV-106}$ (*n*=5), ${bla}_{TEM-1}$ (*n*=5), ${bla}_{OXA-1}$ (*n*=5) and *mcr-1* (*n*=2).

**Fig. 5.**
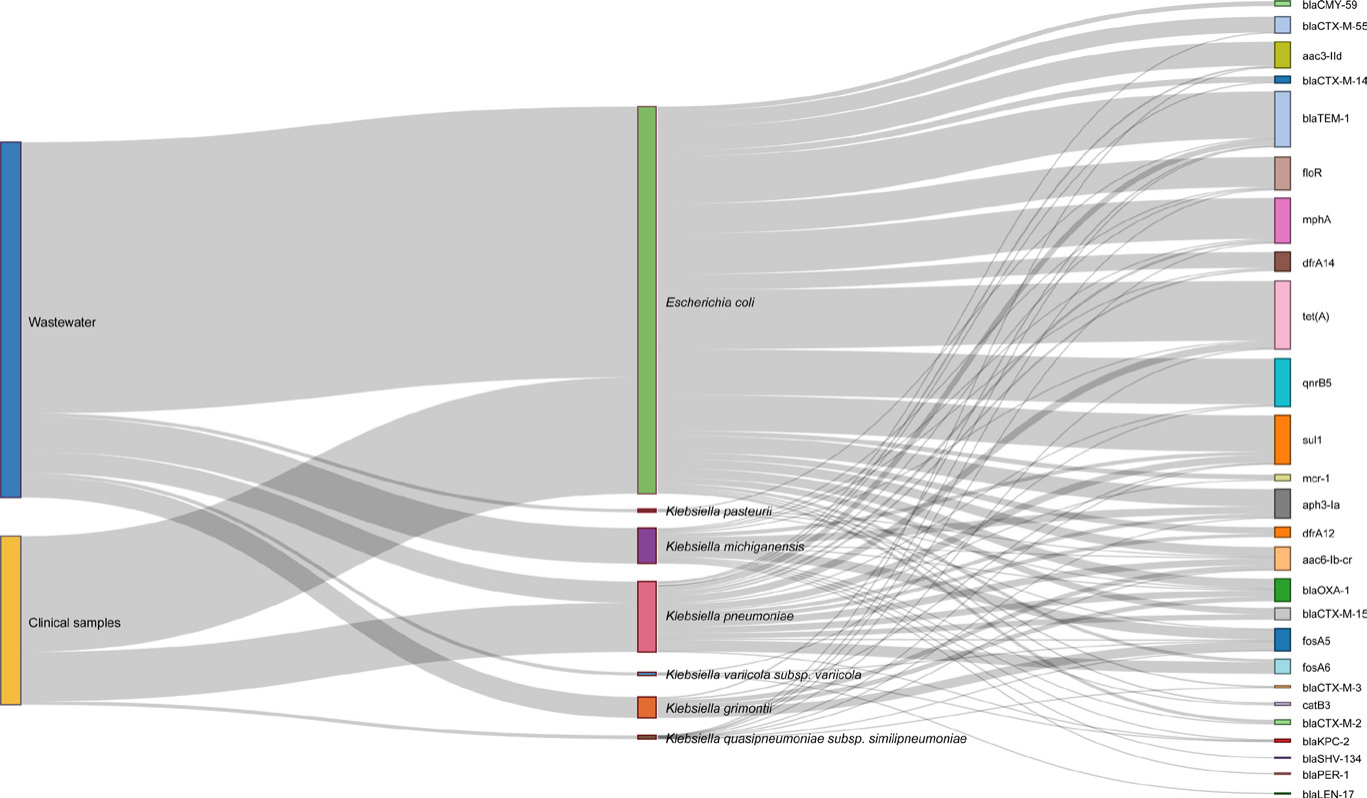
Distribution of the most frequent ARGs by source and species. A Sankey diagram illustrates the associations between sample origin (hospital wastewater vs. clinical isolates), bacterial species (*E. coli* and *Klebsiella* spp.) and the most frequently detected ARGs. The width of the connections represents the frequency of ARGs within each species and source.

The distribution of ARGs varied between clinical and wastewater isolates, with wastewater samples harbouring a broader diversity of ARGs across *E. coli* and *Klebsiella* spp., including genes associated with *β*-lactam resistance (${bla}_{CTX-M-14}$ and ${bla}_{CTX-M-15}$), aminoglycoside resistance (*aac3-IId*), and colistin resistance (*mcr-1*) (Fig. 5). As shown in the Sankey diagram, clinical isolates exhibited a higher prevalence of *β*-lactamase genes, while wastewater isolates displayed a more diverse array of resistance genes, suggesting distinct selective pressures between hospital and environmental settings.

## Discussion

This study provides a genomic and phenotypic characterization of *E. coli* and *Klebsiella* spp. recovered from hospital wastewater and clinical samples at a paediatric hospital in Lima, Peru. We integrated WGS and phenotypic resistance testing to identify diverse resistance profiles and STs across both environments. These findings contribute to understanding how hospital effluents may act as reservoirs of AMR with implications for surveillance strategies in LMICs.

ESBL production was significantly more frequent in clinical *E. coli* isolates than in wastewater isolates, consistent with the strong selective pressures of antibiotic use in healthcare settings [40]. Conversely, carbapenemase-producing isolates were detected only in wastewater, underscoring the potential risk of highly resistant organisms entering the environment through hospital effluents [41]. The detection of the *mcr-1* gene, associated with colistin resistance, in clinical isolates, although limited in number, highlights the circulation of resistance to last-resort antibiotics [42]. Together, these observations reinforce the need for both hospital-based infection control and environmental mitigation strategies.

The identification of 59 STs among *E. coli* and 16 STs among *Klebsiella* spp*.* highlights the high genetic diversity present in clinical and environmental samples. *E. coli* ST-10, one of the most frequently reported STs in hospital wastewater, was among the dominant sequence types identified in this study [43,44]. In contrast, *E. coli* ST-131 and ST-101 were predominant in clinical isolates, consistent with their global association with hospital-acquired infections [45,46]. Notably, the shared presence of ST-88 and ST-101 in both wastewater and clinical isolates suggests a potential link between hospital environments and wastewater, reinforcing the utility of wastewater surveillance in tracking antimicrobial resistance. Among *Klebsiella* isolates, the predominance of *K. pneumoniae* ST-15 and its association with multiple resistance genes (${bla}_{SHV-106}$, ${bla}_{TEM-1}$ and ${bla}_{OXA-1}$) raises concerns about the persistence of highly resistant clones within hospital settings. ST-15 has been implicated in outbreaks of carbapenem-resistant *Klebsiella* in healthcare facilities, emphasizing the importance of ongoing monitoring for this sequence type [47,49].

The presence of overlapping sequence types, such as *E. coli* ST88 and ST101, in both wastewater and clinical isolates suggests potential overlap between bacterial populations in these settings. While no high-resolution SNP analysis was performed, these findings support the hypothesis that hospital effluents may contribute to maintaining or amplifying AMR diversity. Phylogenetic clustering further showed that specific wastewater and clinical isolates grouped closely despite different origins, consistent with possible bacterial flow between the two environments. Moreover, the higher Shannon diversity index observed in wastewater isolates compared to clinical isolates emphasizes the complexity of AMR reservoirs outside direct clinical settings [10,14].

Several limitations must be considered when interpreting these findings. Clinical isolates were restricted to diagnostic urine and blood cultures from paediatric ICU patients, excluding carriage isolates that may represent additional resistance reservoirs. The lack of contemporaneous sampling between wastewater and clinical isolates limits temporal comparisons and precludes direct inference of transmission. Furthermore, the single-hospital focus constrains generalizability to other healthcare or community settings in Peru. Future studies incorporating longitudinal sampling, carriage isolates from patients and healthcare workers, and high-resolution genomic analyses will be critical to clarify potential transmission dynamics.

Despite these limitations, our study demonstrates the value of combining clinical and environmental genomic surveillance to monitor AMR. Wastewater-based monitoring is particularly relevant in LMICs, where routine clinical sequencing may be limited but effluent sampling can provide a broader view of ARG circulation. Establishing integrated surveillance systems that include hospital wastewater could enable earlier detection of high-risk lineages, inform infection control interventions and ultimately help reduce the spread of MDR organisms within healthcare facilities and into surrounding communities.

## Supplementary material

10.1099/acmi.0.001006.v3 Uncited Table S1.

## Abbreviations

AMR: antimicrobial resistance
ARGs: antimicrobial resistance genes
CC: clonal complex
ESBL: extended-spectrum *β*-lactamase
ICU: intensive care unit
LMICs: low- and middle-income countries
MDR: multidrug-resistant
NCBI: National Center for Biotechnology Information
STs: sequence types
WGS: whole-genome sequencing
